# 1250. Association Between Antimicrobial Stewardship Resources and Practices Reported by California Hospitals in the National Healthcare Safety Network Annual Survey and California Department of Public Health Antimicrobial Stewardship Program Honor Roll Designation

**DOI:** 10.1093/ofid/ofad500.1090

**Published:** 2023-11-27

**Authors:** Lizette Brenes, Becca Czerny, Tisha Mitsunaga, Erin Epson, Jane Kriengkauykiat

**Affiliations:** California Department of Public Health (CDPH), Richmond, California; California Department of Public Health, Oakland, California; California Department of Public Health, Oakland, California; California Department of Public Health, Oakland, California; HAI Program, California Department of Public Health, Richmond, California

## Abstract

**Background:**

California hospitals report information on antimicrobial stewardship (AS) Core Elements, resources, and practices through the National Healthcare Safety Network Annual Survey. The California Department of Public Health has an AS Program (ASP) Honor Roll to recognize and promote hospital AS. We explored associations between AS-related responses in the Annual Survey with Honor Roll designation, which ranks increasingly comprehensive ASP as Bronze, Silver, or Gold.

**Methods:**

We linked 2020–2021 Annual Survey data to Honor Roll designations for acute care and critical access hospitals by year. We included 59 AS-related survey questions based on completeness and consistency between survey years. We compared survey responses by designations with chi-square and Fisher’s exact tests. We used a multivariable proportional odds regression model to evaluate the association of survey responses with the likelihood of ordinal designations. We used a stepwise approach and compared goodness-of-fit statistics at each iteration of model variable selection.

**Results:**

Of 179 Honor Roll hospital designations with linked survey data, 21% were Gold, 41% Silver, and 38% Bronze in 2020, and 21% were Gold, 53% Silver, and 26% Bronze in 2021. The final ordinal model included 29 survey covariates related to leadership oversight, ASP lead, AS practices, and education. Hospitals significantly associated with higher designation included those that: performed prospective audit and feedback (PAF) of vancomycin (OR:4.09, 95%CI 1.27-13.83) and azoles (OR:3.37, 95%CI 1.07-11.31); and required prior authorization of newer beta-lactam/-lactamase inhibitors (OR:3.31, 95%CI 1.18-9.49) and echinocandins (OR:2.78, 95%CI 1.02-7.77), holding constant all other variables. Odds for higher designations were reduced if facilities performed PAF of echinocandins (OR:0.17, 95%CI 0.05-0.53).

**Conclusion:**

Higher Honor Roll designation is a marker for a more effective ASP; thus, AS practices, especially prospective audit and feedback, in conjunction with resources related to ASP lead(s) and leadership oversight, should be prioritized by ASPs. Further exploration of practices, such as prior authorization of select antimicrobials, would be warranted as there were varying associations with designations.

**Disclosures:**

**All Authors**: No reported disclosures

